# Discovering Associations of Adverse Events with Pharmacotherapy in Patients with Non-Small Cell Lung Cancer Using Modified Apriori Algorithm

**DOI:** 10.1155/2018/1245616

**Published:** 2018-04-23

**Authors:** Wei Chen, Jun Yang, Hui-Ling Wang, Ya-Fei Shi, Hao Tang, Guo-Hui Li

**Affiliations:** ^1^Department of Pharmacy, National Cancer Center/Cancer Hospital, Chinese Academy of Medical Sciences and Peking Union Medical College, Beijing 100021, China; ^2^Information Management Center, National Cancer Center/Cancer Hospital, Chinese Academy of Medical Sciences and Peking Union Medical College, Beijing 100021, China

## Abstract

**Aim:**

To explore the associations between adverse events and pharmacotherapy in patients with non-small cell lung cancer.

**Methods:**

16,527 patients with non-small cell lung cancer admitted to the Cancer Hospital, Chinese Academy of Medical Sciences, between January 1, 2010, and December 31, 2016, were included in the study. Their medication and laboratory examinations data were extracted from the medical records. Common Terminology Criteria for Adverse Events Version 4.03 were utilized for adverse events reporting. A new association algorithm was developed based on Apriori algorithm and used to investigate the associations between drugs and adverse events. In addition, a statistical comparison was conducted to compare the modified Apriori algorithm with the conventional Apriori algorithm.

**Results:**

Different types and levels of adverse events were identified from the abnormal laboratory findings. The three most common adverse events were hypocalcemia, elevated creatine phosphokinase, and hypertriglyceridemia. In addition, using the modified Apriori algorithm, 380 association rules were found between adverse events and chemotherapy. Moreover, the statistical comparison of the two methods demonstrated that the modified Apriori algorithm was more advantageous in analyzing the correlation between drugs and adverse events than the conventional Apriori algorithm.

**Conclusions:**

The modified Apriori algorithm can be used to more efficiently associate pharmacotherapy with adverse events. Based on the modified Apriori algorithm, meaningful association rules between drugs and adverse events were found, demonstrating a promising way to reveal the risk factors of adverse events during cancer treatment.

## 1. Introduction

Lung cancer is the most common cause of cancer-related death in China. There were 705,000 (470,000 male and 225,000 female) new cases of lung cancer and 569,000 patients (387,000 male and 282,000 female) died of lung cancer in 2012 [1]. The five-year survival rate of lung cancer patients is only 15% [2]. Non-small cell lung cancer (NSCLC) accounts for more than 85% of all lung cancer cases [3]. Pharmacotherapy, especially chemotherapy, is the main strategy for cancer treatment because of its demonstrated efficacy in reducing tumor progression and improving overall survival in advanced cancer patients. However, these treatments frequently cause severe adverse reactions and induce unexpected outcomes, preventing them from being used as first-line therapies [4]. To achieve better long-term prognosis, cancer patients are often treated with combined chemotherapy. However, simultaneous administration of multiple drugs may increase the adverse drug reactions (ADR) [5]. Due to the high prevalence of NSCLC, ADR related to chemotherapy are becoming an increasingly important issue. The use of association algorithms, such as Apriori algorithm, has shown their feasibility and effectiveness in detecting adverse drug events (ADE) [6]. Apriori algorithm was first presented in 1994 [7] and has been widely used for frequent itemset mining and association rule learning [8]. Data mining techniques like Apriori algorithm typically focus on positive association rules based on frequently occurring itemsets to extract association rules from big data. Therefore, these algorithms may ignore many important but infrequent itemsets [9]. In addition, because these algorithms lack attention to the concept and meaning of items, the results may include many nonsense and redundant ones [10].

In this study, we proposed a modified Apriori algorithm to overcome the deficiencies of conventional Apriori algorithm, especially for ADR detection, and studied the relationship of administered drugs with adverse events.

## 2. Materials and Methods

### 2.1. Data Source

The study was approved by the Ethics Committee of Cancer Institute and Hospital, Chinese Academy of Medical Sciences. The database was obtained from the medical records of NSCLC patients who were admitted to Cancer Hospital, Chinese Academy of Medical Sciences, from January 1, 2010, to December 31, 2016. Patients were excluded if they did not complete the therapeutic protocol or had incomplete records. Patients' information including demography, prescription, medical test orders, and results of clinical laboratory tests were extracted and normalized. The collected medical dataset contains the records of 17,048 patients. Every drug and clinical laboratory test was defined as an independent variable and coded for analysis. The collected data were organized using SQL server 2012 database software.

### 2.2. Data Cleaning and Standardizing

The obtained data were streamlined. First, data cleaning was implemented to remove duplicate records in the database. Second, consistency checking was performed to check whether the data meet the requirements and identify data that are beyond the normal range or logically unreasonable. Due to errors in inputting, coding, and extracting, the dataset contains some invalid data and missing data, which were identified by consistency checking. The preferred method for consistency checking was manual retrieval. If manual retrieval was impossible, four other steps could be selected: estimation, case deletion, variable deletion, and pairwise deletion. For a dataset with a small percentage of invalid or missing data, these cases were generally selected for deletion. After data streamlining, 521 (3.06%) patients were excluded and 16,527 patients were included in the study. Among them, 16,527 patients were enrolled in the study meeting data integrity requirements. Of all the studied patients, a total of 1,820,207 prescriptions were extracted from electronic medical record, of which 1,201,594 prescriptions were related to pharmacotherapy. Drugs are classified according to the active ingredients. Drugs with different dosage, forms, or specifications, but with the same active ingredients, are defined as the same type of drug. A total of 8,867,853 clinical test records were extracted from electronic medical record database, involving 502 testing items. The abnormal ones, above or below normal laboratory testing range, were more meaningful and stored separately in the database, including 888,805 above normal and 683,225 below normal tests.

### 2.3. Demographic Data

#### 2.3.1. Age and Gender

**Figure 1:**
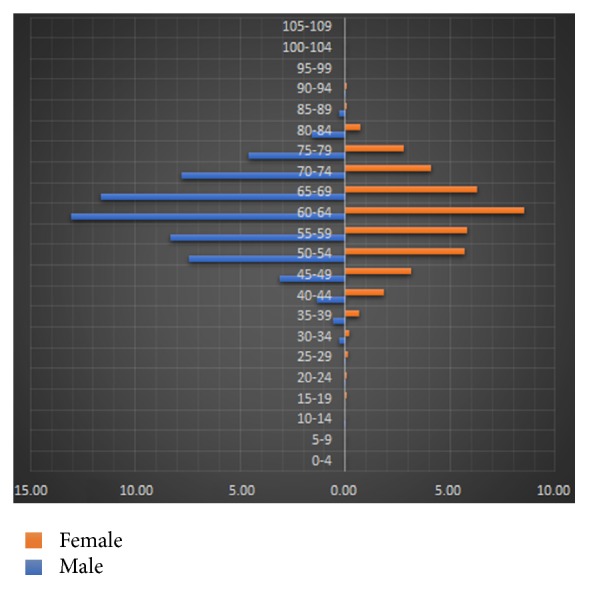
Percentage of patients with different gender and in different age groups in all 16527 enrolled NSCLC patients.

The enrolled 16,527 patients were categorized based on their age and gender. As shown in Figure 1, 9,941 (60.15%) were males and 6,586 (39.85%) were females and the ratio of males to females was 1.51 : 1. Patients were 61.67 years old on average, ranging from 13 to 94 years old at diagnosis and had a median age of 62 years old. The largest population of males and females in the enrolled NSCLC patients was at the ages of 60–64 years.

#### 2.3.2. Geographical Distribution of Patients

**Figure 2:**
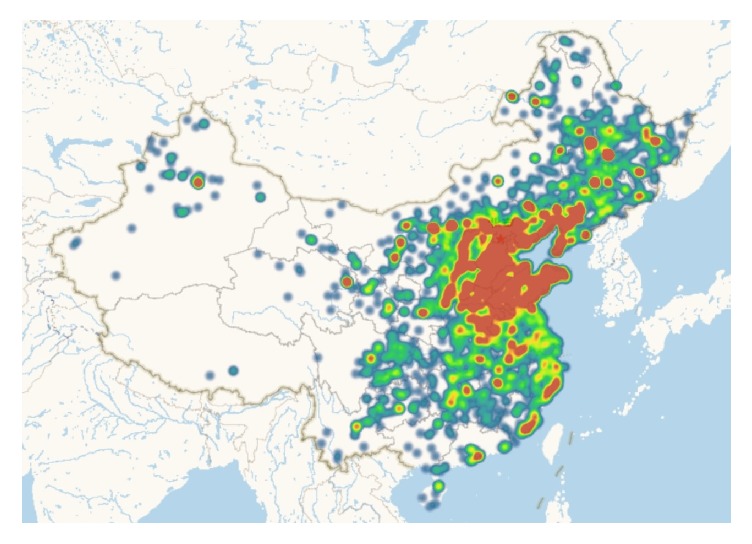
Heatmap of geographical distribution of the patients.

Their street addresses were concealed to protect patient privacy and their district addresses were extracted and converted to latitude and longitude data using the open platform of Baidu maps (http://lbsyun.baidu.com/). Heatmap with superimposed colors was plotted to describe the population distribution (Figure 2).

### 2.4. Algorithm Design

A modified Apriori algorithm was developed by introducing the mechanism of *χ*^2^ for analyzing the positive and negative association rules of administered drugs with clinical test results.

The modified Apriori algorithm for association rule learning can be characterized using 2 steps. Let itemset *I* = {*i*_1_, *i*_2_,…, *i*_*m*_} be a set of items *i*, where the number of items in *I* is *m*. Let transaction *T* = {*t*_1_, *t*_2_,…, *t*_*n*_} be a set of itemsets, where every itemset that *T* contains is a subset of *I*. Let *D* be a set of transactions. Step 1 contains 4 algorithms of conventional Apriori algorithms [8].  Algorithm 1 is the whole pseudocode of Apriori algorithm for screening large itemsets without introducing the mechanism of *χ*^2^.  Algorithm 2 takes the number of first pass items to screen the large itemsets.  Algorithm 3 uses the join subset of large itemsets *L*_*k*−1_ to generate candidate itemsets *C*_*k*_.  Algorithm 4 makes the process more efficient by deleting *C* from *C*_*k*_ if the subsets of *C* do not belong to *L*_*k*−1_. The occurrence of each *C* belonging to *C*_*t*_ is counted. When the result is greater than the minimum support* (min_sup)*, *c* will be added to *L*_*k*_. The algorithm will keep running until *L*_*k*_ is empty.

In order to compensate the deficiency of the original algorithm, step 2 was performed by introducing *χ*^2^ into the algorithm. *χ*^2^ indicates the degree of deviation between the observations and the theoretical values.

It is assumed that there are two categorical variables, *X* and *Y*, whose values are {*x*_1_, *x*_2_} and {*y*_1_, *y*_2_}, respectively. The sample frequency series are shown in Table 1:

(1) χ2=ad−bc2×na+bc+da+cb+d,df=1.

When *n* is greater than 40 and the theoretical frequency of each group is not less than 5, it conforms to *χ*^2^(1) distribution. In this way, the correlation of the two items can be identified according to the testing theory in statistics. The null hypothesis *H*0 is that *X* and *Y* are independent. The argument to be inferred is *H*1 that “*X* is related to *Y*.” At the given significant level *α* = 0.05, if the calculated result is greater than 3.841 (*χ*^2^(1), Chi-square test critical table), the null hypothesis is rejected. Thus, *X* and *Y* are not independent from the statistical point of view at confidence of 0.95. If the calculated result is less than 3.841, the null hypothesis is true. In other words, *X* and *Y* are independent.

Although the original algorithm can examine whether two items are related, it could not distinguish positive and negative association rules. In order to exhibit negative association rules and to implement the modified Apriori algorithm more conveniently, a new screening variable minimum test value* (min_tev)* was defined and calculated by the total sample number and critical value that has been determined by significant level and degree of freedom. Variable* comp* was defined as the degree of positive and negative association by removing square count and holding the sign

(2) min_tev=xα21,comp=nad−bca+bc+da+cb+d.

After Step 1 was applied to screen every possible combination of administered drugs, the* confidence*(*a*/(*a* + *b*)),* comp* of each new drug combination, and each test result were figured out in Step 2. Each new drug combination with* confidence* greater than the thresholds* (min_cof)* was retained.* Comp* greater than* min_tev* indicates positive association rules, while* comp* less than negative* min_tev* indicates negative association rules. In addition, the intensity of association rules can be measured by the absolute* comp* value.

The desired algorithm and statistical analysis were implemented by using* MATLAB* software. All statistical tests were performed at significance level of 0.05.

## 3. Results and Discussion

### 3.1. Treatments

Chemotherapy, surgery, radiotherapy, and interventional therapy were used for treatment of NSCLC. Among them, surgery was the predominant procedure, which was used in 12,804 (77.47%) patients, followed by chemotherapy for 5,122 (30.99%) patients, radiotherapy for 1,777 (10.75%) patients, and interventional therapy for 56 (0.34%) patients. Multimodality therapy was performed for 2,875 (17.39%) patients and single modality therapy was performed for 13,231 (80.04%) patients.

A total of 592 drugs were given to patients including chemotherapeutic agents, analgesics, biologics, antimicrobial agents, glucocorticoid, traditional Chinese medicine, and others. A total of 5,122 patients were treated with different regimens of chemotherapy using 33 types of drugs due to different conditions. Among them, 4,716 (92.07%) patients were treated with multiple drugs and 406 (7.93%) patients with single agent. Among these drugs, platinum-based drugs such as cis-platinum, carboplatin, nedaplatin, oxaliplatin, and lobaplatin played critical roles in chemotherapy and were found in medical orders for 4,767 patients, accounting for 93.07% of all patients treated with chemotherapy. Combination of platinum-based drugs and pemetrexed was the primary regimen, which was used for 2,582 (50.41%) patients, followed by combination of platinum-based drugs and paclitaxel, which was used for 1,451 (28.33%) patients. Single-agent regimen was rarely used. Of them, pemetrexed disodium was administrated in 102 (1.99%) patients and cis-platinum in 97 (1.89%) patients, respectively.  Table 2 shows the most commonly used chemotherapeutic regimens involving one platinum-based drug and another drug as well as their application in patients.

The targeted therapy of tumors has been widely accepted. Among all the patients studied, 782 patients received targeted treatments. Among the 12 involved targeted drugs, Rh-endostatin and bevacizumab, both of which are monoclonal antibodies inhibiting angiogenesis, were the most and second most frequently used drugs, treating 242 and 233 patients, respectively. The third mostly used targeted drug was gefitinib, treating 130 patients.  Table 3 lists the usage of targeted drugs.

### 3.2. Clinical Laboratory Results

**Figure 3:**
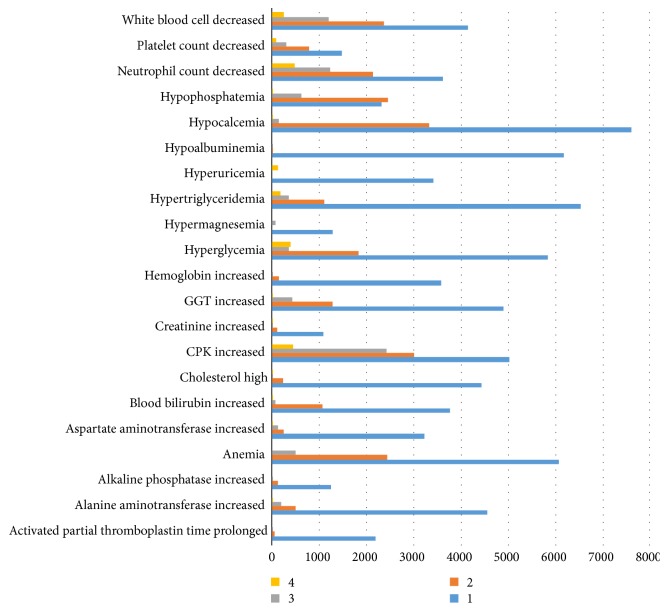
Adverse events with incidence number greater than 1000.

According to the Common Terminology Criteria for Adverse Events (CTCAE) Version 4.03, all different types and levels of adverse events were screened and the number of involving patients was counted. Among all adverse events, 8,915 patients had hypocalcemia, accounting for 53.94% and ranking the first; 8,415 had elevated creatine phosphokinase (CPK), accounting for 50.92% and ranking the second; 6,849 patients had hypertriglyceridemia, accounting for 41.44%; and 6,549 patients had hyperglycemia, accounting for 39.63%. In addition, anemia, hypoalbuminemia, increased GGT, and decreased white blood cell were also important adverse events.  Table 4 lists the number of patients with different types and levels of adverse events and Figure 3 shows the adverse events with more than 1000 incidence.

### 3.3. Comparison of the Two Methods for Data Mining

To obtain all possible association rules that make sense, given the low usage of certain oncology medications and the high incidence of adverse events,* min_sup* was set at 165, which is 1% of the total number of patients, and* min_cof* was set at 10%. In addition, to compare the differences between the modified and conventional Apriori algorithms, the association rules were mined under the same parameters using the two algorithms, respectively. For anticancer drugs, the conventional Apriori algorithm was implemented on MATLAB platform.

The running time of the conventional Apriori algorithm was 35.97 s, and a total of 558 association rules were obtained. Among them, 177 were association rules of single drug and adverse events and 381 were association rules of two drugs and adverse events. Traditional Apriori algorithms cannot distinguish positive association rules or negative association rules. Among these 558 association rules, there were a large number of invalid rules, especially indistinct negative association rules. These invalid rules were troubling for the subsequent analysis of valuable association rules.

The running time of the modified Apriori algorithm was 34.49 s, slightly shorter than that of the conventional Apriori algorithm. A total of 380 association rules were obtained, much fewer than that of the conventional Apriori algorithm. Among them, 119 were association rules of single drug and adverse events, 261 were association rules of two drugs and adverse events, 370 were positive association rules, and 10 were negative association rules.  Table 5 compares the results obtained using the two Apriori algorithms statistically. As shown, the operation time of the modified Apriori algorithm is slightly shorter, and the modified Apriori algorithm can distinguish positive and negative association rules. The rank sum test shows a significant difference in the positive association rules obtained using the modified and conventional Apriori algorithms (*P* = 0.0498 for single drug association rules and *P* = 0.0004 for two drug association rules), but no significant difference in the negative association rules obtained using the modified and conventional Apriori algorithms (*P* = 0.2329 for single drug association rules and *P* = 0.9188 for two drug association rules), possibly due to the fewer negative association rules found by the modified Apriori algorithm. It is believed that when more variables are added, the two will show significant differences. The statistical results clearly show that compared with the conventional Apriori algorithm, the modified Apriori algorithm has slightly shorter calculation time and obviously reduced number of invalid association rules and can distinguish the negative association rules; thus, it is more advantageous in mining the correlation between administered drugs and adverse events.

### 3.4. Top Meaningful Association Rules

In order to find out whether the use of those drugs for tumor treatment can lead to adverse events more easily and which drugs can reduce the incidence of adverse events, the modified Apriori algorithm was used to analyze the correlation of all drugs with adverse events and identify more meaningful association rules. The positive association rules imply that the use of these drugs could lead to adverse events more easily. One should pay attention to their clinical use and, if necessary, preventive and intervention measures.  Table 6 lists the 16 top meaningful positive association rules that cover common adverse events such as hematopoietic system suppression, abnormal liver function, and hyperglycemia.

Decreases in both anemia and neutrophil counts are myelosuppressive side effects of chemotherapy. The incidence of anemia is also high in patients undergoing chemotherapy [11]. Many patients have to undergo other treatments due to chemotherapy-induced anemia [12]. In this study, 38.88% of the enrolled patients exhibited different degrees of anemia. Data mining results show that cisplatin is related to anemia. Decreased neutrophil count is a common side effect of chemotherapy and it is the primary reason for dose delay or decrease [13]. Cisplatin is obviously associated with decreased neutrophils and white blood cells, increased cholesterol and GGT, and hypertriglyceridemia, but it is an important chemotherapy drug for the treatment of NSCLC. Hepatotoxicity could increase the levels of ALT and AST. It has been reported that liver toxicity is a common side effect of pemetrexed-containing regimen [14, 15]. Our results also showed strong correlations between pemetrexed and increased ALT as well as AST, revealing the liver toxicity of pemetrexed. Gemcitabine is associated with decreased platelet count. In addition to chemotherapeutic drugs, the association rules also show that other medications are associated with adverse events. For examples, Folium Sennae is associated with increased blood bilirubin; zoledronic acid is associated with increased alkaline phosphatase; and pazufloxacin is associated with hypermagnesemia. Although Apriori algorithm can reveal the association of drugs with adverse events, it could not reveal the causal links. These association rules still need to be further confirmed using evidence-based medical or experimental studies.

Table 7 lists the top meaningful positive association rules between 5 combinations of medications and adverse events. Among all the patients in the study, 6,426 (38.88%) suffer from anemia. The incidence of anemia is 84.09% in patients using both gemcitabine and carboplatin. The combination of gemcitabine and cisplatin increases the incidence of neutropenia to 73.27%, indicating that it is necessary to pay close attention to the neutrophil count in combination chemotherapy. Association analysis suggests that the combination of paclitaxel and gemcitabine and the combination of nedaplatin and pemetrexed greatly increase alanine aminotransferase (ALT) and aspartate aminotransferase (AST), suggesting they may have addition effects and need to be further studied. Both single-agent and combination chemotherapy can promote hyperglycemia [16]. Hyperglycemia can induce related complications but also attenuate the antiproliferative effects of chemotherapy, as reported by a preclinical study [17]. Among all the patients in the study, 6,549 (39.63%) suffer from hyperglycemia, and 393 (2.38%) have level 4 hyperglycemia. The combination of paclitaxel and carboplatin is associated with the occurrence of hyperglycemia. Almost half (49.72%) of the patients treated with paclitaxel and carboplatin have hyperglycemia.

The modified Apriori algorithm can help distinguish and filter out clinically significant negative association rules, which suggest that these drugs may reduce the occurrence of adverse events in cancer therapy. The discovery of these rules provides clues for further exploration of the mechanisms of drug actions and the development of adjuvant therapies for cancer chemotherapy. Meanwhile, if the adverse events reducing effects of these drugs were confirmed through a higher level of evidence-based medical evidence, these drugs can be useful in clinical applications, directly benefiting cancer patients.

Among those with negative association rules to chemotherapy-induced adverse events, administering antitumor drug disodium cantharidinate and vitamin B6 is negatively associated with decreased white blood cells, neutrophil, and platelet as well as increased AST, indicating that they could be used to fight against cancer but also have no adverse impact on patients' hematopoietic system and liver function and even could increase white blood cells and decrease AST. The published pharmacological action of disodium cantharidinate confirms that injection of disodium cantharidinate with vitamin B6 could increase white blood cells. Further association analysis shows that lentinan is negatively associated with decreased platelet count. The incidence of decreased platelet count is 10.52% in all patients, but only 5.91% in patients treated with lentinan. Lentinan is also negatively associated with decreased neutrophil count. The incidence of decreased neutrophil count is 26.41% in all patients, but only 16.52% in patients treated with lentinan.

## 4. Conclusions

Introducing the Chi-square test into the conventional Apriori algorithm produces a more efficient and accurate algorithm for analyzing the association rules between drugs and their related adverse events in 16,527 medical records. Using the modified Apriori algorithm, drugs associated with adverse events are identified among many association rules. These associations suggest that, in the treatment of NSCLC, one should pay more attention to these drugs and, if necessary, conduct retrospective or prospective clinical studies. In addition, the study shows that cantharidinate in combination with vitamin B6 exerts antitumor effect but also has no adverse effect on hematopoietic system and liver function, suggesting that the combination of these drugs may offer effective and low toxic treatment. Therefore, their anticancer mechanisms need to be further studied. Although many rules are obtained through the modified association algorithm, these rules only reveal the correlation and mutual exclusion between them. The intrinsic causality and mechanism of action need to be further studied.

## Figures and Tables

**Algorithm 1:**
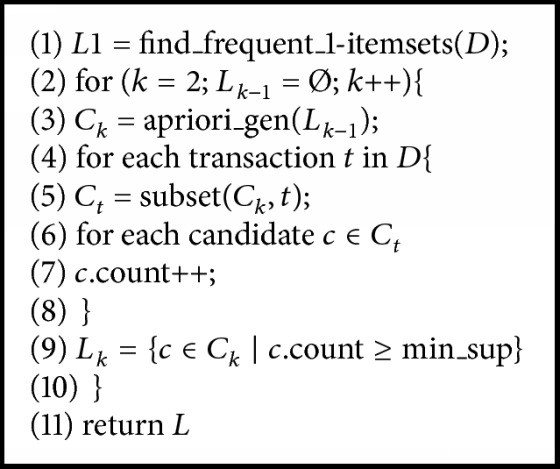
The pseudocode of finding frequent itemsets.

**Algorithm 2:**
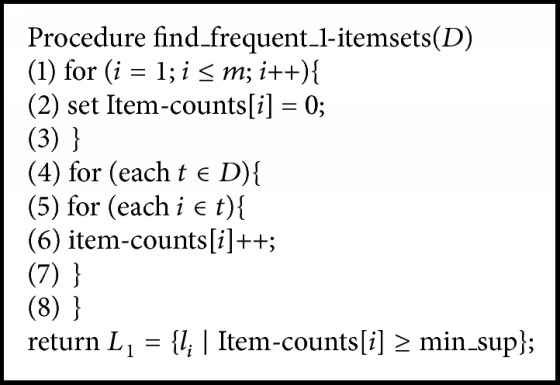
The pseudocode of finding frequent 1-itemsets.

**Algorithm 3:**
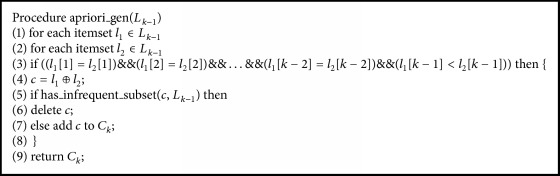
The pseudocode of join.

**Algorithm 4:**
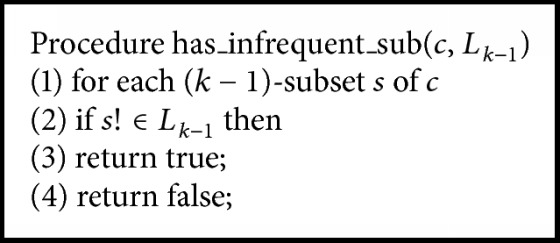
The pseudocode of prune.

**Table 1:** Series of sample frequency.

	*y*_1_	*y*_2_
*x*_1_	*a*	*b*
*x*_2_	*c*	*d*

**Table 2:** Different chemotherapy regimens used for advanced cancers.

Agents	Number of patients	Percentage of all chemotherapy patients
Platinum-based drugs and pemetrexed	2,582	50.41%
Platinum-based drugs and paclitaxel	1,451	28.33%
Platinum-based drugs and gemcitabine	807	15.76%
Platinum-based drugs and docetaxel	574	11.21%
Platinum-based drugs and etoposide	189	3.69%
Platinum-based drugs and vinorelbine	33	0.64%

**Table 3:** Targeted drugs used in treatment.

Agents	Number of patients	Percentage of patients treated with molecular targeted drugs
Rh-endostatin	242	28.01%
Bevacizumab	233	26.97%
Gefitinib	130	15.05%
Erlotinib	74	8.56%
Icotinib	63	7.29%
Nimotuzumab	53	6.13%

**Table 4:** The number of patients with different types and levels of adverse events.

SOC	Adverse event	1	2	3	4	Grand total	Percentage (%)
Blood and lymphatic system disorders	Anemia	6076	2439	512		6426	38.88%
Investigations	Activated partial thromboplastin time prolonged	2203	59	8		2222	13.44%
Investigations	Alanine aminotransferase increased	4567	498	201	20	4762	28.81%
Investigations	Alkaline phosphatase increased	1250	132	28		1283	7.76%
Investigations	Aspartate aminotransferase increased	3230	249	131	24	3366	20.37%
Investigations	Blood bilirubin increased	3777	1083	76	8	4165	25.20%
Investigations	Cholesterol high	4430	242	7	4	4459	26.98%
Investigations	CPK increased	5023	3006	2421	455	8415	50.92%
Investigations	Creatinine increased	1091	110	7	2	1135	6.87%
Investigations	Fibrinogen decreased	413	78	19	6	475	2.87%
Investigations	GGT increased	4896	1283	427	21	5286	31.98%
Investigations	Hemoglobin increased	3577	157	9		3603	21.80%
Investigations	Lymphocyte count decreased	292	188	68	8	362	2.19%
Investigations	Neutrophil count decreased	3616	2133	1241	485	4364	26.41%
Investigations	Platelet count decreased	1487	795	316	97	1738	10.52%
Investigations	White blood cell decreased	4143	2364	1195	252	5134	31.06%
Metabolism and nutrition disorders	Hypercalcemia	646	22	18	9	669	4.05%
Metabolism and nutrition disorders	Hyperglycemia	5832	1844	362	393	6549	39.63%
Metabolism and nutrition disorders	Hypermagnesemia	1285		87		1348	8.16%
Metabolism and nutrition disorders	Hypertriglyceridemia	6532	1117	361	179	6849	41.44%
Metabolism and nutrition disorders	Hyperuricemia	3414			133	3438	20.80%
Metabolism and nutrition disorders	Hypoalbuminemia	6183	1	1		6183	37.41%
Metabolism and nutrition disorders	Hypocalcemia	7598	3332	154	21	8915	53.94%
Metabolism and nutrition disorders	Hypoglycemia	254	16	2	1	269	1.63%
Metabolism and nutrition disorders	Hypomagnesemia	396	9	5		404	2.44%
Metabolism and nutrition disorders	Hyponatremia	85		32	8	115	0.70%
Metabolism and nutrition disorders	Hypophosphatemia	2328	2470	639	15	4384	26.53%
Renal and urinary disorders	Proteinuria	673	47			707	4.28%

**Table 5:** Comparison in the data mining speed and number of association rules generated by using the conventional and modified Apriori algorithms.

	Conventional Apriori algorithm				Modified Apriori algorithm				Comp Rank sum test
Data mining speed	35.97				34.49				
Number of association rules	558				380				
	Number	Comp average	Support average	Confidence average	Number	Comp average	Support average	Confidence average	
Association rules of single drug and adverse events									
Positive association	129	10.932	0.083	39.562	110	12.654	0.084	41.033	0.050
Negative association	48	−6.348	0.111	28.932	9	−8.183	0.156	11.021	0.233
Association rules of two drugs and adverse events									
Positive association	314	6.829	0.025	41.278	260	8.042	0.027	42.926	0.000
Negative association	67	−3.567	0.031	30.073	1	−3.356	0.085	11.373	0.919

**Table 6:** Meaningful association rules between adverse events and drugs.

Adverse event	Drug	Comp	Support	Confidence
Anemia	Cisplatin	26.897	0.174	61.426
Activated partial thromboplastin time prolonged	Meropenem	15.757	0.036	36.500
Alanine aminotransferase increased	Pemetrexed	21.411	0.162	46.197
Alkaline phosphatase increased	Zoledronic acid	20.635	0.043	28.212
Aspartate aminotransferase increased	Pemetrexed	16.549	0.162	32.327
Blood bilirubin increased	Folium Sennae	8.834	0.196	31.459
Cholesterol high	Cisplatin	13.373	0.170	37.834
GGT increased	Cisplatin	16.731	0.172	45.753
Hemoglobin increased	Lentinan	4.793	0.235	25.064
Neutrophil count decreased	Cisplatin	46.431	0.173	61.619
Platelet count decreased	Gemcitabine	25.428	0.051	37.470
White blood cell decreased	Cisplatin	39.577	0.173	62.929
Hyperglycemia	Diprophylline	12.320	0.038	63.535
Hypermagnesemia	Pazufloxacin	17.857	0.015	38.889
Hypertriglyceridemia	Cisplatin	16.980	0.170	56.539
Hyperuricemia	Cisplatin	11.228	0.168	29.501

**Table 7:** Meaningful association rules between adverse events and drug combination.

AE	Clinical laboratory	Drug	Comp	Support	Confidence
Anemia	HGB	Gemcitabine + carboplatin	13.74	1.33%	84.09%
Neutrophil count decreased	NEUT#	Gemcitabine + cisplatin	15.58	1.31%	73.27%
Alanine aminotransferase increased	ALT	Paclitaxel + gemcitabine	8.19	1.08%	56.74%
Aspartate aminotransferase increased	AST	Nedaplatin + pemetrexed	6.65	1.23%	39.22%
Hypoglycemia	GLU	Carboplatin + paclitaxel	6.16	5.42%	49.72%
